# The Name.Narrate.Navigate (NNN) Program: A Case Study of Tertiary Intervention for Justice-Involved Youth in Regional Australia

**DOI:** 10.3390/bs16050679

**Published:** 2026-04-29

**Authors:** Tamara Blakemore, Louise Rak, Susan Rayment-McHugh, Elsie Randall, Chris Krogh, Meaghan Katrak Harris, Sally Hunt, Daniel Ebbin, Graeme Stuart, Shaun McCarthy

**Affiliations:** 1 Centre for Violence, Prevention and Healing, University of Newcastle, Callaghan, NSW 2308, Australia; louise.rak@newcastle.edu.au (L.R.); chris.krogh@newcastle.edu.au (C.K.); meaghan.katrakharris@newcastle.edu.au (M.K.H.); sally.hunt@newcastle.edu.au (S.H.); daniel.ebbin@newcastle.edu.au (D.E.); graeme.stuart@newcastle.edu.au (G.S.); shaun.mccarthy@newcastle.edu.au (S.M.); 2 Sexual Violence Research and Prevention Unit, School of Law and Society, University of the Sunshine Coast, Sippy Downs, QLD 4556, Australia; srayment@usc.edu.au; 3 Justiz Community, Mayfield, NSW 2304, Australia; elsie@justiz.com.au

**Keywords:** youth violence, trauma-informed practice, culturally responsive intervention, realist evaluation, tertiary intervention, dialectical behaviour therapy, photovoice

## Abstract

Name.Narrate.Navigate (NNN) is a trauma-informed program for justice-involved young people aged 12–18 years, recognising that experience and use of violence are often interconnected and may involve serious criminal behaviour, including vulnerability to criminal exploitation. NNN addresses a gap in evidence-based, culturally responsive tertiary interventions for this cohort in regional New South Wales (NSW), Australia, integrating dialectical behaviour therapy (DBT) principles with Aboriginal ways of knowing and doing, co-designed through community-based participatory research (CBPR) with Aboriginal community members, young people, and frontline practitioners. The program aims to strengthen skills for self-awareness, self-regulation and healthy connection through relational, creative, and participatory approaches. Using a realist evaluation framework, this paper examines what works in NNN, for whom, and under what circumstances. Drawing on participant session ratings, practitioner observations, program documentation, and interviews, findings are organised across four domains: effects, mechanisms, moderators, and implementation. Indicative findings show that engagement, emerging changes in the narratives of self, and developing skills for self-regulation were most evident when trauma-informed and culturally safe practice was enacted within genuinely relational, strengths-based encounters. These conditions are identified and discussed as transferable principles for the field, key amongst them that intervention readiness must be treated as a capacity to be actively built rather than a precondition to be screened for; and that creative, participant-led methods represent an epistemological commitment to whose knowledge counts in practice. This case study contributes to a critically underserved evidence base by documenting not only what a tertiary youth violence intervention looks like, but the conditions under which it begins to work and for whom.

## 1. Introduction

Youth violence is both familiar and unknown. Familiar as a common, perhaps ubiquitous, context of human and social service practice, a constant focus of media reporting and political positioning; unknown in that policy and practice have yet to produce approaches that instil sustained confidence in either prevention or response. In 2016, a conversation with a local Children’s Court magistrate crystallised what the authors, as practitioner-academics, knew all too well: that the experiences of young people before the Court for criminal matters are a complex study of contrast. These children have commonly both experienced and caused harm, the latter often overshadowing the former to an outsider’s gaze (Levenson & Willis, 2019). They are also at once known and unknown, their actions often highly visible and visceral across media platforms, but largely unheard in systems and settings that determine their fate (Fasulo et al., 2015, Moore & McArthur, 2017). In practice, this can translate to them being both agents of power and control and vulnerable citizens who may be marginalised and oppressed—commonly having protracted histories with child protection and justice systems yet remaining disengaged from—and actively disengaged by—education and support systems.

In an effort to better understand these experiences, we began with a small pilot study (Blakemore et al., 2018, 2019), exploring practitioner narratives about the justice-involved young people in regional New South Wales (NSW), Australia. Semi-structured interviews with 37 practitioners across policing, juvenile justice, physical and mental health, child protection and out-of-home care, education and Aboriginal-specific services revealed accounts that were strikingly context-bound. Practitioners spoke about young people’s involvement in crime with a firm eye on location, socioeconomic and sociocultural experience, relational transactions and interactions they felt shaped both violence and its meaning. They described youth violence as becoming more frequent and intense, particularly for young women (Blakemore et al., 2018) and were frank about what they saw as the inadequacy of the existing service landscape to respond in trauma-informed and culturally responsive ways. Further, practitioners noted appropriate training for them to work in these ways was neither accessible nor affordable. To us a parallel process seemed to be at play—young people disengaged with and from systems; and workers disenfranchised by systems without the right services or scaffolds for strengthening capacity (Blakemore et al., 2018, 2019). This structural and relational parallel became the conceptual seed of the Name.Narrate.Navigate (NNN) program.

This paper is a practice-informed case study using a realist evaluation lens—not a formal outcome evaluation, and not a claim of definitive program effectiveness. It proceeds in six sections. We begin by examining the impetus and context for NNN, before describing the community-based participatory design process through which NNN was developed. A description of the program itself follows, including its theoretical foundations, content, and structure. We then outline the realist evaluation methodology used to assess the program’s mechanisms and outcomes, before presenting findings organised through the EMMIE framework—Effects, Mechanisms, Moderators, Implementation, and Economic Value (Johnson et al., 2015). The paper concludes with implications for tertiary youth violence intervention more broadly, with particular attention to the relational and culturally grounded ‘new way’ of working that the NNN program represents.

## 2. Impetus and Context

The impetus and context of this case study are intertwined. The location of our work is characterised by both considerable strengths and challenges. While not unique among regional Australian contexts, parts of the region include communities with entrenched and compounding patterns of socioeconomic disadvantage, including persistently elevated rates of unemployment and youth unemployment, and school completion rates that remain significantly below state and national averages (Australian Bureau of Statistics, 2021; NSW Department of Education, 2024; Vinson & Rawsthorne, 2015). For Aboriginal young people in particular, these challenges occur within a broader context of ongoing colonisation, the enduring consequences of the Stolen Generations, and a service system not always experienced as culturally safe or responsive. High rates of child protection reports, out-of-home care (OOHC), and domestic and family violence (DFV) in the region further contextualise it as one in which children and families face significant and intersecting pressures (NSW Department of Communities and Justice, 2019; BOCSAR, 2025).

The region is also recognised as an epicentre of historic institutional child sexual abuse (McPhillips, 2018). Consistent with evidence on the intergenerational impacts of institutional child sexual abuse, including diminished trust in, and engagement with, institutions offering health, education and support services (Royal Commission into Institutional Responses to Child Sexual Abuse, 2017), a number of communities across the region demonstrate elevated rates of psychosocial distress and comparatively poor engagement with preventive health and education services (Hunter New England and Central Coast Primary Health Network, 2022).

Despite these challenges, the region has demonstrated sustained capacity for renewal across successive periods of adversity, including recovery from Australia’s most destructive earthquake in 1989 and adaptation to what has been described as the nation’s largest deindustrialisation event following the closure of BHP’s Newcastle steelworks in 1999 (Atteridge & Strambo, 2021). The Institute for Regional Futures (2023) at the University of Newcastle, suggests this capacity for adaptation over time has been strengthened by the region’s standout levels of social capital and community resilience, characteristics documented empirically as giving Hunter communities particular advantage during periods of significant socioeconomic transition. The human and social service workforce reflects this—large, relatively stable, and staffed by skilled and committed employees with longstanding ties to the community (Blakemore et al., 2018, 2019).

A significant outcome of the pilot study (Blakemore et al., 2018, 2019), was a collective recognition amongst those involved that this region—precisely because of its characteristic contexts—was well-placed to develop community-driven, ground-up solutions to working with youth violence and that the relevance of such work might extend well beyond its local origins. That conviction is borne out by the broader evidence that despite decades of research interest effective tertiary interventions for young people engaged in the most serious and persistent violent offending remain critically scarce, an issue made particularly pressing given what the data tells us about the scale and complexity of the problem.

Both then, and now, NSW crime data has confirmed that police are increasingly responding to youth violence. In the year October 2024–September 2025, 6036 young people aged 10–17 years were proceeded against by police for offences including domestic violence-related assaults (*n* = 1202), intimidation, stalking and harassment (*n* = 1938), murder (*n* = 9), non-domestic violence-related assault (*n* = 2677), sexual assault (*n* = 115), sexual touching, and sexual acts and other sexual offences (*n* = 95) (BOCSAR, 2026). Young people charged with these offences represented almost 30% of the 20,802 young people proceeded against by police for criminal behaviour in the previous year, a number that has increased by 59.1% in the ten years to September 2025 (BOCSAR, 2026).

Within this broader trend, the gendered dimensions of youth violence have become increasingly complex. Historically, the majority of all young people proceeded against by police for the violence-related charges have been reported as male and their victims often female (Freeman, 2018). However, between 2014 and 2023 the number of young females aged 10–17 yrs proceeded against by police grew by 20% compared to relatively stable trends for young men (Donnelly, 2024). Despite a notable decline during the COVID-19 period, from 2022 on “the number of offences committed by young females increased and exceeded prior yearly volumes of offending for this cohort” (Donnelly, 2024, p. 8). Comparison of the offence rates of young men and young women aged 10–17 years in NSW from 2014 to 2023 found 14.7% of all female offenders were charged with non-domestic violence-related assault, 9.3% with domestic violence assault, and 7.6% with intimidation, stalking and harassment (Donnelly, 2024). By comparison, 7.5% of all male offenders were charged with non-domestic violence-related assault, 5.2% with domestic violence assault, and 6.4% with intimidation, stalking and harassment (Donnelly, 2024).

While the minimum age of criminal responsibility (MACR) across most states and territories in Australia is 10 years of age, the majority of young people proceeded against by police are aged between 14 and 17 years (Freeman & Donnelly, 2024). This largely reflects application of *doli incapax* which presumes a young person under 14 years lacks capacity to be criminally responsible for their actions. A rebuttable presumption, *doli incapax* places the onus on the prosecution to prove the young person both committed the criminal act and knew what they were doing was morally wrong, rather than in the legal formulation being “merely naughty or mischievous”^1^. Despite this, in 2023, the NSW police initiated legal proceedings against 4662 young people aged 10–13 years, one third of which were for violence-related offences (Freeman & Donnelly, 2024). It is notable that 41.3% of these young people were Aboriginal and the rate of legal proceedings against regional/rural and remote young people under 14 years was more than three times higher than those from major cities (Freeman & Donnelly, 2024). The operation of *doli incapax* in NSW has itself become a site of active policy reform. An independent review commissioned by the NSW Government (Bellew & Loy, 2025) found diversion is likely the most appropriate response for less serious offending for young people aged 10–13 years, and in late 2025 NSW passed legislation codifying and strengthening the *doli incapax* test—improving prosecutors’ ability to rebut the presumption and giving courts clearer guidance on the circumstances of the alleged offending (NSW Government, 2025). Notably, this policy reform differs in ethos and intent from other Australian jurisdictions, including the ACT and Victoria, which have moved to raise the minimum age of criminal responsibility for this cohort to 12 or 14 years (Bellew & Loy, 2025).

The crime statistics presented capture the use of violence reported and recorded according to offence types. In practice, young people’s use of violence encompasses a broader range of physical and non-physical behaviours including assault, intimidation, property damage, coercive control, financial abuse and psychological abuse—acts not always aligned to the offence types discussed above, and some not yet captured within the current NSW legislative framework. For example, while NSW has recently introduced coercive control legislation, its application to young people remains an evolving area of policy and practice (Wangmann, 2024). Even with appropriate legislation in place, it is well established that youth violence, particularly within the family, is significantly under-reported (Fitz-Gibbon et al., 2018; Donnelly, 2024), meaning the statistics presented here are likely to underrepresent the true prevalence of young people’s use of violence in their relationships and communities.

Crime statistics also fail to represent the reality that young people’s relationship with violence is rarely one-dimensional. Research consistently shows that many who use violence have also experienced it, often forming what is now recognised as a ‘crossover cohort’, young people whose histories of child protection involvement are deeply entangled in their subsequent use of violence toward others. In Australia, Baidawi and Sheehan (2019) have found that young people with prior child protection contact are significantly more likely to engage in persistent and serious offending, with crossover histories compounding risk in ways that neither the child protection nor the youth justice system has been adequately designed to address. This pattern is further evidenced in South Australian contexts (Malvaso et al., 2019, 2021), and is nowhere more starkly evident than for Aboriginal and Torres Strait Islander young people, who are profoundly overrepresented within this cohort. Despite only making up 6.6% of the Australian youth population, Aboriginal young people are detained at a rate 21 times their non-Aboriginal peers, representing 60% of all incarcerated youth (Australian Institute of Health and Welfare, 2025), with child protection systems involved in the lives of approximately half of those in detention (Papalia et al., 2020).

For Aboriginal young people, the use and experience of violence cannot be understood apart from its colonial origins. The violent dispossession of land and culture, the state-sanctioned removal of Aboriginal children across generations, and the structural racism embedded in Australian institutions have produced enduring aftershocks of trauma, disconnection and marginalisation (Barnes & Motz, 2018; Cunneen & Tauri, 2019). These aftershocks are actively compounded by present-day involvement with child protection and youth justice systems that were not designed for, nor adequately adapted to the intersecting needs of this cohort, and in which interventions intended to support best interests can instead compound the very harms they seek to address (Menzies, 2019; J. Miller & Berger, 2020). The Royal Commission into the Protection and Detention of Children in the Northern Territory (2017), prompted by the exposure of systemic abuse of young people, the vast majority of them Aboriginal, at the Don Dale Youth Detention Centre, documented how institutions charged with the care and rehabilitation of children can themselves become sites of harm. Its findings underscored that the consequences of colonisation and structural racism are not only historical but are actively reproduced through the very systems young people are referred to for help (Royal Commission into the Protection and Detention of Children in the Northern Territory, 2017).

### Existing Approaches to Youth Violence Intervention

Understanding what NNN is requires understanding what it is not—and specifically, what the existing landscape of intervention offers, and where it falls short for the cohort of young people this paper concerns. Notable programs for youth violence in Australia have included anger management (Howells et al., 2005), aggression replacement training (Glick et al., 2011), multisystemic family therapy (Henggeler et al., 2009), and Step-Up (Routt & Anderson, 2016), most drawing predominantly on cognitive-behavioural principles. While these have demonstrated effectiveness with some populations, the evidence base remains limited and conflicting (Boxall et al., 2020; Fitz-Gibbon et al., 2022). CBT-based approaches assume a relatively direct relationship between thinking, feeling, behaviour and change—and with it, a degree of cognitive accessibility and readiness for engagement—that complex trauma and structural disadvantage routinely undermine (Howells & Day, 2003; Lipsey, 2007, 2009). For young people whose use of violence is shaped by those very conditions, the fit between the assumptions on which CBT is based and the contextual reality of their experience is significantly constrained.

A related limitation is that few existing interventions were designed to engage with the intersecting dynamics of trauma, marginalisation, and cultural dislocation that characterise the experiences of some young people involved in serious offending (Campbell et al., 2020; Moulds & Day, 2017). For Aboriginal young people in particular, this gap can be acute. Violence cannot be understood apart from its colonial contexts—the dispossession of land and culture, the intergenerational consequences of child removal, and the ongoing experience of systems that have compounded rather than addressed harm (Atkinson, 2002; Dudgeon et al., 2014). Programs that do not engage with these realities are unlikely to be experienced as culturally safe by the young people they are designed to reach.

Most critically, effective tertiary interventions for young people most deeply involved in serious and persistent violent offending—and particularly those whose histories reflect compounding involvement with child protection, justice and other systems—remain the least developed and least evidenced tier of the continuum (Campbell et al., 2020; Boxall et al., 2020; Fitz-Gibbon et al., 2022; Hollonds, 2024). Research consistently identifies criminogenic needs—including deficits in emotional literacy, communication skills, empathy and the capacity to understand and navigate power, control, and shame—as drivers of violence in young people (Andrews & Bonta, 2010). From a strengths-based perspective (Saleebey, 2012; Ward & Maruna, 2007), these are not fixed ‘deficits’ but capacities—context-bound and multi-determined in their development and expression, but capable of growth and change through targeted intervention. For young people at the most serious end of the continuum, this reframing—from deficit to capacity, from driver to target—is an important basis for meaningful engagement with the possibility of change toward desistance (Maruna, 2001; Ward & Maruna, 2007).

NNN was developed precisely to address these gaps—in theoretical fit, in cultural responsiveness, and in the evidence base for tertiary intervention—bringing together trauma-informed, culturally responsive, and strengths-based principles in a program co-designed with and for the cohort that existing interventions have most consistently struggled to reach.

## 3. Program Design Process

NNN was developed and has been continuously improved through community-based participatory research (CBPR), a social justice-informed approach involving increasing degrees of active collaboration between researchers and the community (Minkler & Wallerstein, 2003). Building on the pilot work described earlier (Blakemore et al., 2018, 2019), a stakeholder consortium—an advisory group of young people with lived experience, a cultural reference group of Aboriginal community members facilitated by an acknowledged Elder, a practitioner working party and a sector steering committee—was formed to support the design of NNN. With appropriate ethics approvals in place^2^, over the course of the pilot, five meetings were held with each of the consortium groups—the steering committee, practitioner working party, and cultural reference group—moving from feedback on an evidence synthesis through iterative co-design to endorsement of program activities. Through this process, consortium members provided practice and lived experience perspectives, ensured local relevance, challenged assumptions and tested and trialled activities that became part of the program (Rak et al., 2024a).

Foremost among the insights emerging from these consultations was the observation that many young people who use and experience violence are referred to multiple supports yet rarely genuinely engage with them (Blakemore et al., 2019, 2021a). A structural mismatch became clear: service system indicators of engagement—punctuality, self-regulation, being articulate and capacity for polite, constructive client–worker dialogue—were the same capacities complex trauma and disadvantage routinely undermine. Effective service engagement and meaningful change require a certain degree of ‘readiness’ that this cohort, as a result of their circumstances and context, were often without. Practitioners too face their own version of the readiness gap—while committed and often experienced in trauma work, they report feeling underprepared for practice targeting the use of violence, particularly in trauma-informed and culturally responsive ways. The design rationale of NNN was, therefore, not to replace existing casework or specialist intervention, but to address a dual gap in ‘readiness’—building foundational skills in young people and equipping practitioners with tools and scaffolding to work with youth violence in trauma-informed and culturally responsive ways (Rak et al., 2024a).

NNN recognises that foundational skills in self-awareness, self-regulation and connection are critical for young people to achieve positive and sustained change in life circumstances, wellbeing and safety—including cessation of violence. Its theory of change (TOC) acknowledges these foundational skills encompass a broader set of specific ones—including skills to recognise, regulate and communicate emotions and needs, empathy to respond to the self and other, and the capacity to navigate power, control and shame in making choices toward positive change (Rak et al., 2024a). NNN theorises that these skills constitute ‘targets’ of change—best addressed through ‘mechanisms’ that build knowledge and skills, sustained by growing confidence, connection and coping. While oriented toward different outcomes, these targets of change are also relevant to practitioners, who require the same foundational skills—self-awareness, self-regulation and connection—as the basis for genuinely trauma-informed and culturally responsive practice.

## 4. Program Description

NNN was designed for young people aged 12–18 years who have used and experienced violence and the practitioners who support them, toward mutual goals of self-awareness, self-regulation and skills for connection. The program draws on Dialectical Behavior Therapy (DBT) (Linehan, 2015), Kolb’s (2015) experiential learning model, universal design learning (UDL) (Rose & Meyer, 2006), and Photovoice (Fitzgibbon & Healy, 2019; Wang, 1999) to support these goals in trauma-informed and culturally responsive ways.

This case study focuses specifically on NNN’s application as a tertiary intervention with justice-involved young people already engaged in serious and persistent violent offending, including those charged with the most serious offences. Although NNN has since been delivered across a broader range of cohorts and settings, including with young people at earlier points on the risk continuum, that work is not the focus of this paper. With this justice-involved cohort, NNN was delivered in settings that are known and familiar to them, operating alongside, rather than instead of, existing supports including juvenile justice supervision, specialist mental health support and individual casework. The program comprised an individual orientation, six weekly group sessions of approximately 90 min attended by four to six young people and two practitioners, and an individual debrief. Program graduates were invited to contribute to NNN in peer mentoring or consultancy roles, recognising the expertise embedded in their lived experience.

The six group sessions in NNN are sequentially structured around the component skills identified in the TOC as targets of change—emotional literacy, communication skills, empathy, power and control, shame and choice. Activities within sessions deliberately alternate between higher and lower intensity, building capacity for self-regulation in ways consistent with neurodevelopmental principles for trauma-informed practice (Perry, 2009; Warner et al., 2013).

NNN practitioners work from five core practice principles: validation of trauma, reciprocal communication, mindful engagement, shared power and skills for connection (see Table 1). Threading through these principles, under the guidance of Aboriginal Practice Lead and acknowledged Elder Aunty Elsie Randall, is an approach of ‘Deep Listening’—an Aboriginal practice of respectful and reciprocal listening and conversation (Bessarab & Ng’andu, 2010; Brearley, 2014)—adopted to honour young people’s lives and stories, facilitate sensitive conversations about violence, and promote connectedness, learning and self-awareness (Blakemore et al., 2021b).

Each group session is consistently structured around six core components: check in/check out; mindfulness; experiential learning; Photovoice; ‘Postcards to Practice’; and session rating scales. The components (presented in Table 2 and discussed thereafter), do distinct but complementary work: some directly implement mechanisms of change, others create conditions necessary for those mechanisms to operate, and some do both. Their combination is intentional and scaffolded: without the conditions created by some components, the learning activated by others is less likely to take hold and without the mechanism components, the conditions created are not enough to support behavioural change.

Alongside the program for young people, and reflecting the same commitment to self-awareness, self-regulation and connection, is a parallel process of reflective practice for practitioners. Following each session, practitioners complete a structured reflection document that seeks immediate observations about the experience of delivering each core component. From this point, practitioners engage in a reflective conversation with a member of the NNN project team, focused on gathering lessons from implementation, ensuring accountable practice, and identifying any issues that need to be followed up for participants.

Practitioner reflection forms, alongside other anonymous and deidentified data collected in the program—Photovoice images and narratives, Postcards to Practice and session rating scales—are analysed by the project team with the aforementioned ethics approvals and requisite participant consent protocols in place. Importantly, young people can participate in NNN without contributing their work to associated reviews and research. Outcomes of the analysis are shared with the stakeholder consortium, informing ongoing refinement of the program and the development of professional education, training and tools to support practitioners working with youth violence more broadly.

## 5. Realist Evaluation Methodology

In 2021, researchers from the Sexual Violence Research and Prevention Unit at the University of the Sunshine Coast (UniSC) were contracted by the NNN project team to complete an evaluation of the pilot rollout of the program. At this point, NNN had been delivered to 112 young people aged 12–18 years during the period 2018–2021, referred by case managers in justice (*n* = 47) and education staff (*n* = 65). The justice-referred cohort (*n* = 47) comprised roughly equal numbers of male and female identifying participants, with a mean age of 15 years (range 14–18), of whom almost 40% identified as Aboriginal. All had pleaded or been found guilty of violence-related offences and were on community supervision orders at the time of referral. Although NNN has since been delivered across a broader range of cohorts and settings, the evaluation reported here focuses specifically on its original and core application as a tertiary intervention with justice-involved young people; accordingly, findings draw on data from the justice-referred cohort (*n* = 47) only.

The evaluation was undertaken nearing the end of the pilot funding period, with approval from relevant institutional ethics processes.^3^ It took a pragmatic approach—focusing on how the program worked, as well as on implementation and immediate to short-term outcomes for participants and local practitioner stakeholders (Rayment-McHugh et al., 2021). It was founded upon Pawson and Tilley’s (1997) realist evaluation methodology, which seeks to understand program outcomes in terms of both the mechanisms embedded in the program and the context in which it occurs. Rather than asking ‘does this program work, and how effective is it?’, the realist evaluation paradigm asks, ‘for whom does this program work, how, and under what conditions?’ (Rayment-McHugh et al., 2021; Case et al., 2022).

To reduce participant burden the evaluation drew primarily on program-generated materials collected across three timepoints—pre-program, in-program, and post-program (Rayment-McHugh et al., 2021). This included attendance records, Postcards to Practice and Session Rating Scales, as well as data from the orientation and exit sessions. During the pilot phase, two practitioner-administered tools were used in these sessions to build an initial picture of each young person’s world and their communication strengths and areas for growth: one modelled on the Australian Research Alliance for Children and Youth Common Approach to Assessment, Referral and Support (ARACY CAARS) tool (Australian Research Alliance for Children and Youth, 2013) and one on the Strengths and Soft Spots Inventory (Egan, 2010). Program-generated materials were supplemented by two focus groups with five practitioner stakeholders, and semi-structured interviews with three program participants (Rayment-McHugh et al., 2021). The UniSC evaluators used thematic analysis to review qualitative data, with validity checking undertaken by a second member of the evaluation team, and descriptive statistics where quantitative data were available (Rayment-McHugh et al., 2021). To arrange and communicate the findings, four of the five domains of Johnson et al.’s (2015) realist evaluation-informed EMMIE framework were applied—Effects, Mechanisms, Moderators, and Implementation. The fifth domain, Economic Value, was not used as the evaluation did not seek a cost–benefit analysis (Rayment-McHugh et al., 2021). Complementing the UniSC evaluation, thematic analysis of practitioner reflection forms was conducted independently by the NNN project team, providing an additional internal perspective on program delivery and participant experience.

### Limitations

This evaluation was designed to be bespoke to the program, drawing on data sources embedded in NNN’s existing program processes rather than imposing external measurement tools that would place added burden on a vulnerable participant group. As such, its methods and data sources reflect the program’s trauma-informed and participatory commitments as much as its evaluative aims. The robustness of the evaluation was constrained by both the volume and nature of available data: pre- and post-program data were available for eleven of the 34 participants who attended all sessions; the qualitative interview sample was small (*n* = 3) and comprised female participants only, meaning male participants’ experiences are not represented in the interview data; program tools and materials were stretched to an evaluative purpose for which they had only a partial fit; COVID-19 disruptions affected delivery and evaluation; and, as a single-site regional pilot, findings may not transfer directly to other contexts and populations. These constraints mean the findings presented should be taken as indicative and consistent with the action research frame within which NNN operates, and are not considered as endpoints but inputs, continuously feeding back into program refinement, informing subsequent phases of design and delivery, and building an evidence base that grows with the program itself. With these caveats in mind, the findings presented in Section 6 represent meaningful early insights into NNN’s effects, mechanisms, moderators, and implementation—insights that have directly informed later iterations of the program and that warrant further investigation as the evidence base develops.

## 6. EMMIE Findings

The findings that follow have a two-layer structure. The outer layer represents outcomes of the UniSC evaluation (Rayment-McHugh et al., 2021), conducted at one point in time, generating nine indicative findings across four EMMIE domains. The inner layer is drawn from the NNN program’s own research, documented across delivery of the program in a series of publications and an associated book (Blakemore et al., 2024b). Where these two layers speak to the same findings, the program’s own analyses deepen interpretation, illuminating not just that something worked, but why—and what it looked and felt like when it did. Consistent with realist evaluation methodology, findings within each domain are presented as integrated accounts of what was observed, how it operated, and under what conditions, rather than as discrete observations followed by separate interpretation (Pawson & Tilley, 1997; Johnson et al., 2015).

### 6.1. Effects

The UniSC evaluation found that NNN shows promise in promoting positive change among participants. Session rating scale data, collected as part of NNN’s standard program processes and drawn on by the UniSC evaluation, showed young people enjoyed the program and showed active efforts to attend each week. This was seen as different from other programs where young people avoided attending and were reported by their case workers as hard to engage. Consistent effects were seen in NNN’s later delivery at the Frank Baxter Youth Justice Centre (Chand & Blakemore, 2025), where young men in a custodial setting demonstrated comparable engagement and growing self-awareness).

Program participants’ deep need to be listened to came through in the Postcards to Practice data, while focus group information showed that stakeholders saw the program’s commitment to listening to the young people as being a key element to the program and its engagement with young people. More substantively, focus groups with practitioners and interviews with young people noted shifts in personal narrative—including emerging capacities to tell new, nuanced and more reflective stories about their lives and their use of violence. Each of the three young people who completed interviews with the evaluation team was able to describe a changed relationship with violence since participating in the program, with developing knowledge of alternatives to violence evident in the final session’s Postcards to Practice data (Rayment-McHugh et al., 2021).

The UniSC evaluation also found that NNN generated positive benefits for practitioners supporting young people who use violence. Referring and supporting practitioners reported feeling valued as partners and noted NNN’s influence on their own approach—particularly in embedding a more consistent trauma-informed lens in their work with young people (Rayment-McHugh et al., 2021). Beyond engagement, practitioners who support young people involved in the program have reported broader indicators of change for some participants—including those with lengthy histories of serious offending. These include shifts in relationships with family members and caseworkers, re-engagement with education and employment, and reductions in criminal and violent behaviour reported over time. For some graduates, this movement has extended to employment with NNN itself—as peer facilitators and consultants—a form of contribution that reflects not only behavioural change but a fundamental reorientation of identity and purpose. While these outcomes cannot be attributed to NNN alone, and are not captured in formal longitudinal data, they are consistent with the program’s theory of change and with what the mechanisms identified in the evaluation would predict.

Analysis of reflections from NNN practitioners extends this picture, bringing the voices and experiences of young people into sharper focus. Over the course of participation in the program young people are observed to gain confidence to share narratives that refute stereotypes of violence as mindless or unconsidered. Instead, they describe using violence to find and form connection, for acceptance and protection, to communicate needs and to redress injustice—accounts that, while not excusing harm caused, speak to a more complex and constrained form of agency (Blakemore et al., 2024d). Some young women in the program have shared that violence was central to their sense of self and their position in social groups—they felt it necessary to be *known* to have a readiness for violence (Rak et al., 2025). Young people also explained how their violence was contextualised by explicit and implicit experiences of power and control. For example, young people recounted interactions with systems and structures (like school, the police, the court, caseworkers, and mental health) where they used violence as a means of ‘disruptive’ communication in response to being ‘controlled’ (Blakemore et al., 2024d). Experiences of control in the context of relationships seemed less likely to elicit violence—especially for young women who seemed indifferent or resigned to being harassed by family for cash on pay day, or their partner having a ‘right’ to know their whereabouts, who they spend time with, and access to their phone and social media accounts (Blakemore et al., 2024d).

When supported to examine their agency more deliberately, some young people began to contemplate what (positive) change might look like and what it would cost. Considering the positives and negatives of choices toward desistance after his release from detention, Jamal readily named what he stood to lose—money, excitement, belonging—alongside what he stood to gain (Blakemore et al., 2024c). The capacity to hold both realities as simultaneous truths reflects the tension dialectical work in the program navigates between validating a young person’s experience as contextually meaningful while simultaneously making space for the possibility of change (Rak et al., 2024b). For some NNN graduates, this movement has extended beyond the program itself. Jazz, reflecting on her experience, expressed a desire to give something back: “I want to help those kids as much as I can to change, I’ve been in those ways before and I want to see them change, it’s how far I’ve come and I can see where I was then … I enjoy people see how I have changed” (Rak et al., 2024b). Consistent with Maruna’s (2001) account of sustained desistance as rooted in a generative, other-centred orientation, this kind of identity migration—from recipient to contributor—is among the more meaningful indicators of change that the program has observed.

### 6.2. Mechanisms

The UniSC evaluation identified two key mechanisms through which NNN produced its observed effects. The first was the creation of a safe and strengths-based space in which young people felt seen, acknowledged, and valued—for many, an experience that was genuinely novel in their interactions with services (Rayment-McHugh et al., 2021). Sessions were experienced as structured yet responsive, and young people had meaningful agency over their own engagement with the program. The second was the role of creative methods like Photovoice and the practice of Deep Listening in enabling nuanced, multidimensional exploration of complex issues that avoided simplistic binaries and was grounded in the lived geographies of young people’s lives (Rayment-McHugh et al., 2021).

Analysis of NNN practitioner reflections provides examples of both of these mechanisms. A consistent and striking pattern observed across deliveries was that within the group setting young people were contemplative and collaborative—yet outside it, for example during Photovoice excursions, they were louder, more guarded, at times outwardly aggressive, though still notably showing covert care for each other and for practitioners (Blakemore et al., 2024d). The contrast was instructive: the group setting was an active relational container—one in which young people who, in other contexts, could not afford to slow for, or show, reflection or vulnerability, found conditions in which those capacities became accessible. This is consistent with neurodevelopmental understandings of trauma and engagement (Perry, 2009), and with what the evaluation identified as the creation of safety as a precondition for change.

The role of Photovoice as the primary mechanism for creative exploration was evident across all sessions. In addition to its function as a culturally responsive and literacy-inclusive method, Photovoice worked to “speed up” engagement—young people quickly moved into roles as experts on their own experiences, storying their images and educating practitioners in the room (Blakemore & Rak, 2024). Crucially, it also surfaced knowledge that more conventional dialogue-based approaches might not have reached. On one Photovoice excursion, practitioners and young people were followed by police officers who questioned the group, responding to the practitioners’ explanation with scepticism and continuing to trail them. Young people were entirely unsurprised by this, recounting it as a common experience; for practitioners, it was a first-hand encounter with the daily reality of structural suspicion that young people carried into every interaction (Blakemore et al., 2024d). Moments like this gave concrete, shared meaning to the program’s content on power, control, and invalidation—and allowed practitioners and young people to engage with those themes from common ground rather than across a power differential.

The adaptability of creative methods as a mechanism was further demonstrated through NNN’s implementation with incarcerated youth, where restrictions on camera use required Photovoice to be replaced with scaffolded drawing activities facilitated by an embedded design researcher-practitioner (Chand & Blakemore, 2025). Practitioner reflections noted a “creative calm” produced by these activities—participants who were otherwise reactive and guarded became more open, sharing stories and engaging reflectively in ways that parallel findings from community-based delivery (Chand & Blakemore, 2025). That the core mechanism held across this significant adaptation is instructive: it is the creative, embodied, participant-led quality of the method—not Photovoice specifically—that appears to be the operative element.

Deep Listening functioned as a relational thread running through the program. Young people consistently shared narratives that not being heard, and having their feelings and experiences invalidated, was itself a driver of violence (Rak et al., 2024b). They described using violence when invalidated by systems as a form of communication, and when invalidated by peers or family as a way of forming belonging and connection. Deep Listening, as a practice of respectful and reciprocal conversation, offered a direct counter to this experience—and in doing so, addressed one of the structural conditions most likely to undermine engagement before it began.

### 6.3. Moderators

In contrast to the Effects and Mechanisms findings, the Moderator and Implementation findings are less substantive and are primarily drawn from the UniSC evaluation alone. These findings are reported as learnings, noting they have directly informed subsequent iterations of NNN. The evaluation found that participant vulnerability and the complexity of young people’s lives moderated the strength of program effects (Rayment-McHugh et al., 2021). Young people attended NNN while managing complex trauma, unstable housing, and in some cases active family obstruction to their attendance. The depth of this instability is perhaps most starkly illustrated by practitioner reflections on the shared experiences of young women in the program one of whom described custodial detention as a refuge from their outside lives—describing it as ‘the best Christmas ever’, noting the absence of conflict, the shared meal, and the small gift of toiletries (Rak et al., 2025). That detention could be experienced as relief speaks to how severely the between-session world moderated what even a relationally rich, short-term program could offer. COVID-19 disruptions across the pilot period added a further constraint. The degree of support available from external service partners also moderated outcomes significantly—partners provided not only referral and transport but the between-session relational continuity the program alone could not supply, though the resource intensity of this was noted as potentially unsustainable without dedicated support (Rayment-McHugh et al., 2021).

### 6.4. Implementation

The UniSC evaluation identified two foundational conditions for effective NNN delivery. The first was team composition: recruiting practitioners with the specific blend of skills, relational sensitivity, and cultural competence the program requires was identified as essential, with program fidelity seen as inseparable from practitioner quality (Rayment-McHugh et al., 2021). The second was post-program continuity: young people who had developed genuine connections and emerging capacities within NNN needed structured pathways after the program ended—the absence of these was seen as a risk of undermining the gains made (Rayment-McHugh et al., 2021).

Both findings have since directly shaped NNN’s evolving program scope and specifics. Developed from evidence generated by the program for young people, the NNN Practice Pathways program now provides specialist training for cross-sector practitioners across trauma awareness, program delivery, and emerging areas of practice need. Practitioners trained to deliver NNN are offered mentoring as they first deliver the program in their own settings, with opportunities for co-delivery and in situ adaptation support. Post-program mentoring and counselling support pathways for graduates have been formalised—consistent with the program’s broader commitment to support towards identity migration within and across contexts.

The evaluation’s finding that existing pre- and post-program assessment tools were only partially fit for evaluative purpose has also been addressed. New data collection instruments have been adopted—including the Schedule for the Evaluation of Individual Quality of Life–Direct Weighting (SEIQol-DW) (Chenhall et al., 2010), a conversationally driven measure used effectively with justice-involved Aboriginal youth, and the *Now.See.Hear!* tool, a photo-card and conversational prompt instrument co-designed through extensive consultation with young people and practitioners to explore emotional recognition, empathy, prosocial reasoning, and self-narratives in trauma-informed and culturally safe ways (Blakemore et al., 2025).

Implementation learning has also extended to questions of setting and adaptation. Delivery of NNN with incarcerated young men demonstrated that the program can be delivered effectively in custodial contexts, where specific adaptations were required—most notably, the substitution of Photovoice excursions with scaffolded drawing activities. Young men who participated in this delivery were engaged, demonstrated growing self-awareness, and participated with what practitioners observed as an unusual generosity in sharing their stories. Notably, the NNN way of working was observed to produce a distinctive calm in the group space—a quality that practitioners reflected may have value not only within sessions but in reducing tension in the custodial environment more broadly (Chand & Blakemore, 2025). At the same time, the custodial context introduced distinctive implementation challenges: the unpredictability of participant availability, the presence of detention staff, and the resource requirements of an embedded creative practitioner all point to implementation costs and conditions that require careful planning in correctional settings (Chand & Blakemore, 2025).

Across all implementation contexts, the centrality of the service ecology surrounding NNN has been a consistent and important learning. External service partners are not peripheral to the program—they are the conditions of its possibility. They provide referral, transport, between-session relational continuity, and the kind of sustained, known-worker support that the program itself cannot supply within its short timeframe. The resource intensity of this role was noted as a vulnerability in the pilot (Rayment-McHugh et al., 2021) and has directly informed NNN’s approach to scale. Rather than requiring dedicated external resourcing, the program has invested in building delivery capacity within existing workforces—training practitioners to deliver NNN in their own settings and adapting the model across modalities including school-based, on Country, and one-to-one delivery. This approach positions the partnership function not as an add-on to be resourced separately, but as a competency to be embedded in the practitioners already present in young people’s lives.

## 7. Implications for Tertiary Youth Violence Interventions

The case study presented of the NNN program offers a documented, theorised and transferable account of the conditions under which a tertiary intervention for the most complex, most persistently underserved cohort begins to work, for whom and in what ways. This case study sits in dialogue with literature describing existing interventions for youth violence and the gaps identified in their theoretical base, reach and responsivity. The indicative rather than definitive nature of the case study findings also reflects the complex realities of this work and the evidence that exists for and from practice. The following implications are offered not as conclusions, but as transferable principles for a field in which effective practice with young people who use, and experience violence remains critically underdeveloped.

### 7.1. Readiness Is a Target, Not a Precondition

The structural mismatch that shaped the design of NNN—that the capacities service systems require for meaningful engagement are the same capacities complex trauma and disadvantage routinely undermine—has important implications for work with this cohort. For young people at the most serious end of the risk continuum, intervention readiness cannot be a precondition to be screened for and waited upon, but a capacity to be actively developed. Programs that begin from an assumption of baseline readiness will consistently fail to reach the young people who most need them. This is not a new observation in the literature, but it remains insufficiently acted upon in the design and commissioning of tertiary intervention. NNN’s positioning—not as a replacement for specialist recidivist programs but as a readiness-building complement to them—offers one model for how this gap might be addressed. Across our deliveries, practitioners have observed indicators of broader change for some participants—including re-engagement with education and employment, more constructive relationships with caseworkers and family members, and reductions in offending reported over time. While these observations are not captured in formal longitudinal data and cannot be attributed to NNN alone, they are consistent with the program’s theory of change and with what the mechanisms identified in the evaluation would predict. Notably, some of these changes have been observed even among young people with lengthy histories of serious offending—suggesting that readiness-building work, when it operates through relational and culturally responsive mechanisms, may generate effects that extend well beyond the program itself.

### 7.2. Creative Methods as a Legitimate Way of Knowing

A consistent finding across NNN’s deliveries has been that creative methods surface knowledge about young people’s lives and their relationship with violence that more conventional dialogue-based approaches cannot reliably reach. Photovoice worked to speed up engagement, position young people as experts in their own experiences, and generate concrete, shared understandings that bridged rather than reinforced the practitioner–participant power differential. Where camera restrictions in custodial delivery required adaptation to drawing activities, the core mechanism held—the creative, embodied, participant-led quality of the method proved more important than the specific technique (Chand & Blakemore, 2025). The implication for the field is that creative methods represent not a delivery preference but an epistemological commitment—a decision about whose expertise is recognised, what kinds of knowledge count, and how the gap between lived experience and professional understanding can be genuinely narrowed. This commitment is not incidental. Story and visual knowledge are central to Aboriginal knowledge systems (Blakemore et al., 2024a), and their integration into NNN reflects a recognition that method and cultural responsiveness are inseparable. For a cohort that is, as this paper’s introduction notes, often highly visible yet profoundly unheard, methods that do not depend on verbal fluency, that are grounded in lived geography, and that invite rather than extract are likely to be both more equitable and more effective. The development of the *Now.See.Hear!* visual conversation tool—co-designed with young people and practitioners to explore the experiences and capacities most relevant to intervention—reflects NNN’s commitment to building assessment and practice infrastructure consistent with these principles (Blakemore et al., 2024a).

### 7.3. What Tertiary Interventions Are Ultimately for

The most meaningful indicators of change observed across NNN’s deliveries have not been behavioural in the narrow sense, but narrative—shifts in the way young people talk about themselves, their experiences, the role of violence in their lives, and what might be possible in their futures. Jazz’s articulation of wanting to help young people like herself characterises the identity migration we began to see as young people moved toward change—from recipient to contributor, from subject of intervention to agent of it. The implication is that tertiary interventions for the most complex cohort need to be oriented not simply toward reducing risk indicators but toward creating conditions in which a different identity becomes imaginable and inhabitable—possible, probable, and permissible. This requires more than a program. It requires post-program pathways, sustained relational support, and a service ecology that can hold young people as they navigate the often difficult journey of identity migration. It also requires a field willing to invest in a new way of working and a new kind of evidence base for and from practice. NNN’s contribution to that project is the beginning of a conversation about what practice with young people who use and experience violence can look like.

## Figures and Tables

**Table 1:** NNN practice principles.

Practice Principle	What It Means in NNN	Why It Matters
Validation of trauma	Practitioners explicitly acknowledge the role of trauma in young people’s lives while holding the consequences of violence separate	Young people who use violence are often pathologized rather than understood in context; validation creates the psychological safety necessary for engagement (Linehan, 2015; Wall et al., 2016)
Reciprocal communication	Practitioners share their own felt experience and perspective alongside young people rather than positioning themselves as experts	Mirrors the relational conditions under which trust and learning are possible for young people whose experiences of systems have been invalidating (Linehan, 2015; Turney, 2012)
Mindful engagement	Practitioners and young people engage simultaneously with activities with awareness, curiosity and without judgement	Trauma disrupts self-awareness and self-regulation; mindful engagement builds the capacity to notice and respond rather than react (Linehan, 2015; Evans-Chase, 2015)
Shared power	Young people are positioned as experts in their own lives with agency over their experience of the program	Counteracts experiences of systemic powerlessness that often drive violence in this cohort (Campbell et al., 2020)
Skills for connection	Building knowledge, skills and behaviours for improved safety, wellbeing, and relationships	Violence can impact skills needed for relationships—emotional recognition and regulation, communication, empathy, understanding of power and control, and the capacity to navigate shame and make positive choices (Lipsey, 1992; Tangney et al., 2007).

**Table 2:** Program components and their purpose.

Component	Implements Mechanism of Change	Creates Conditions for Change	Brief Explanation
Check in/ Check out		✓	Enacting the practice principle of reciprocal communication, young people and practitioners rate their emotional state against visual emoji cues. Consistent with (UDL) principles (Rose & Meyer, 2006), this component is inclusive of all learning styles and literacy levels, reducing barriers to participation.
Mindfulness	✓	✓	Enacting the practice principle of mindful participation, young people and practitioners practice observatory, descriptive and participatory mindfulness to build self-awareness and self-regulation. Drawn from DBT principles (Linehan, 2015), repeated practice in a safe relational context can support the neurological foundations for engagement and change (Perry, 2009; Warner et al., 2013).
Experiential learning	✓		Activities focused on target skills involve active, embodied engagement rather than didactic instruction. Consistent with Kolb (2015), this recognises that developing and strengthening skills for emotional literacy, communication, empathy, power, control, shame and choice is most effective when learning is active, reflective and grounded in experience—particularly for young people whose experience has been shaped by trauma and disadvantage.
Photovoice/ Photovoice review	✓	✓	Enacting all practice principles, young people and practitioners undertake photo excursions, creating images that are then used as the basis for reflection and discussion within sessions. A culturally responsive method that is not reliant on written language (Castleden et al., 2008), Photovoice grounds learning in the lived experience and geography of young people’s lives, making abstract concepts concrete and personally meaningful.
Postcards to Practice		✓	Enacting the practice principles of reciprocal communication and shared power, young people anonymously respond to open-ended prompts (e.g., ‘If you spent a day in my shoes, you would know…’), in written or drawn form at the end of each session. Postcards to Practice give young people a structured, low-pressure channel to communicate their experiences back to practitioners, ensuring their voice actively shapes the knowledge base for practice.
Session rating scales		✓	Enacting the practice principle of shared power, young people anonymously rate sessions against four criteria: whether they felt heard, whether content was relevant to them, whether they liked the activities, and an overall rating (S. D. Miller & Duncan, 2000; Duncan et al., 2003). Ratings are reviewed and actively inform subsequent delivery.

## Data Availability

Data are unavailable due to privacy and ethical restrictions relating to the protected nature of the young people involved.

## Notes

1 *doli incapax* is discussed in the High Court case of RP v The Queen [2016] HCA53.

2 Ethics approvals were obtained from the Human Research Ethics Committees of the Australian Institute of Aboriginal and Torres Strait Islander Studies (AIATSIS), the University of Newcastle (UON), and the NSW Departments of Education and Justice.

3 See Note 2.
